# Supplementary material for: Anti-Cancer Effects of α-Cubebenoate Derived from Schisandra chinensis in CT26 Colon Cancer Cells
Source: Molecules. 2022 Jan 23;27(3):737. doi: 10.3390/molecules27030737 (PMC8839175; doi:10.3390/molecules27030737)
Supplement: Supplementary file 1 [file molecules-27-00737-s001.zip › molecules-1487504-supplementary.pdf]

Supplement Figures

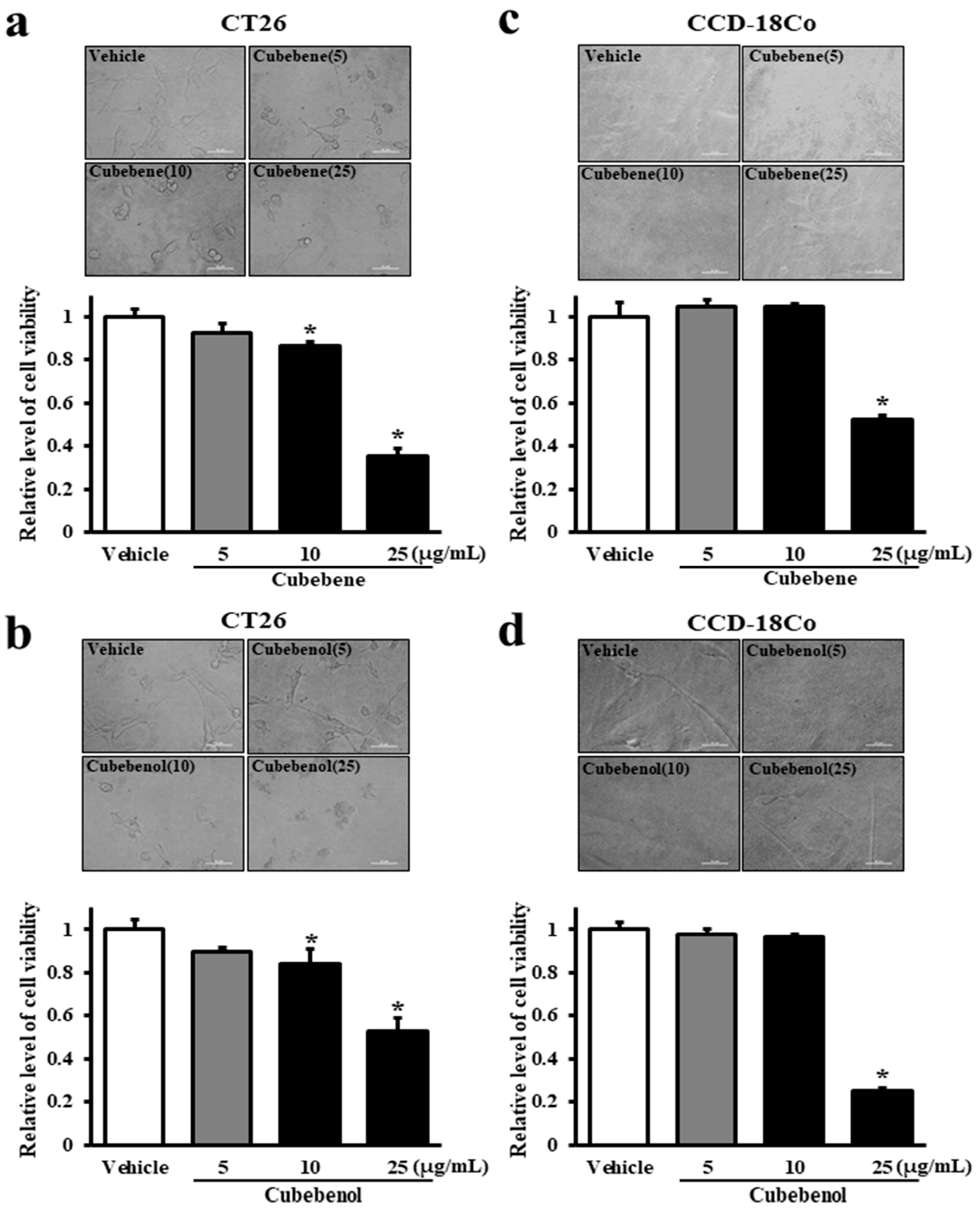

Supplement Figure S1. Cytotoxicity of cubebene or α-iso-cubebenol treated CT26 cells (a and b) and CCD-18Co (c and d). After incubation of CT26 and CCD-18Co cells with 5, 10 and 25 μg/mL of cubebene or α-iso-cubebenol for 24 h, the morphological changes of cells were observed under a microscope at 200× magnification. The cell viability was analyzed with MTT assay. Two to three wells per group were used in the MTT assay, and optical density was measured in duplicates. Data are reported as the means ± SDs. *, $p < 0.05$ relative to the Vehicle treated group.

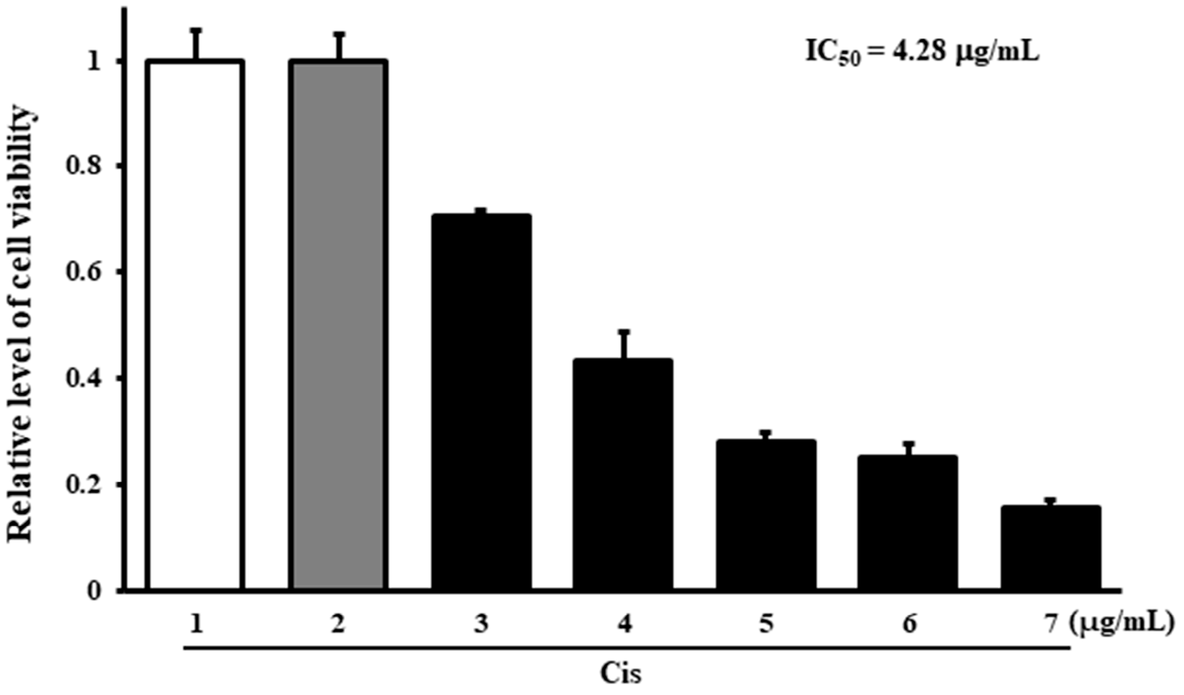

Supplement Figure S2. Determination of optimal concentration for Cis treatment. After incubation of CT26 cells with 1, 2, 3, 4, 5, 6, and 7 μg/mL of Cis for 24 h, the cell viability was measured using MTT assay. Two to three wells per group were used in the MTT assay, and optical density was measured in duplicates. Data are reported as the means ± SDs.

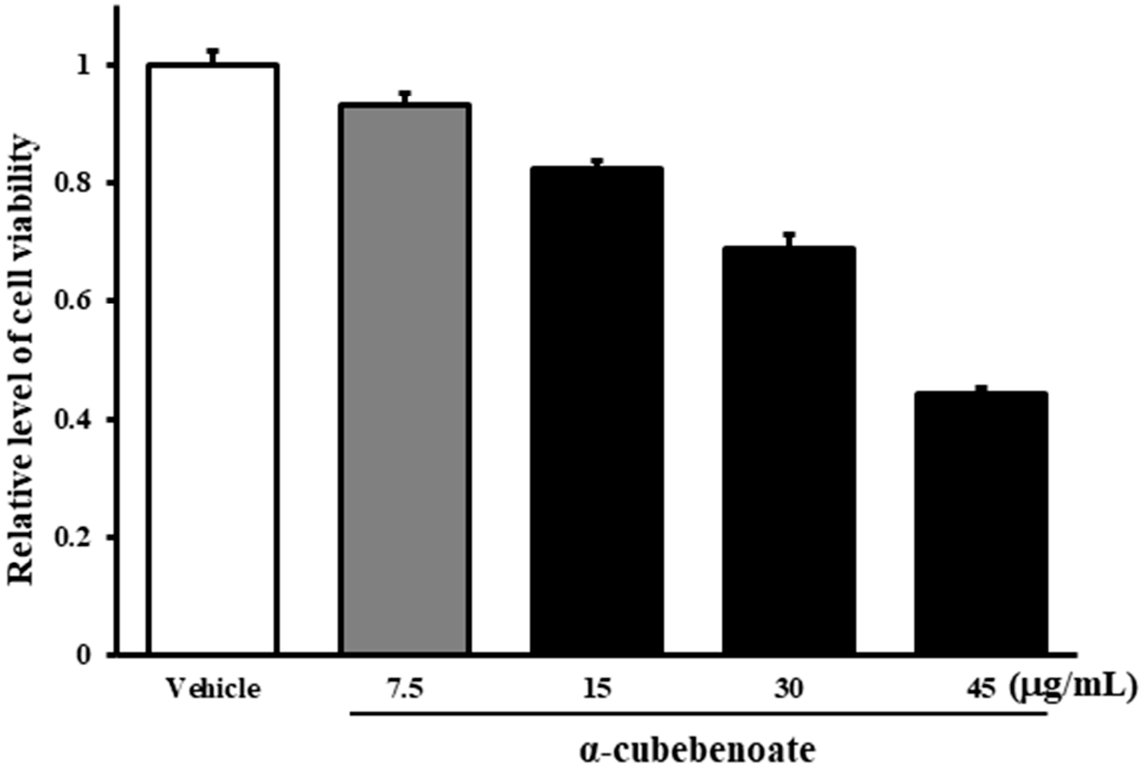

Supplement Figure S3. Determination of optimal concentration for α-cubebenoate treatment. After incubation of CT26 cells with 7.5, 15, 35, and 45 μg/mL of α-cubebenoate for 24 h, the cell viability was measured using MTT assay. Two to three wells per group were used in the MTT assay, and optical density was measured in duplicates. Data are reported as the means ± SDs.
